# Cardiovascular Risk Assessment Among People With Type 2 Diabetes Mellitus in Urban Slums of Central Karnataka, India

**DOI:** 10.7759/cureus.46687

**Published:** 2023-10-08

**Authors:** Shubha Davalagi, Rohit Amuje, Shalini H

**Affiliations:** 1 Community Medicine, Jagadguru Jayadeva Murugarajendra (JJM) Medical College, Davangere, IND

**Keywords:** qrisk3, risk assessment, urban slums, type 2 diabetes mellitus, cardiovascular risk

## Abstract

Background: The intricate interplay between type 2 diabetes mellitus (T2DM) and cardiovascular diseases (CVD) necessitates a comprehensive investigation into the cardiovascular risk landscape among individuals with T2DM. The burgeoning global burden of both conditions underscores the urgency of targeted research in this area, with the potential to inform preventive strategies and mitigate adverse cardiovascular outcomes. By unravelling the risk of CVD among T2DM patients and identifying key risk factors, the current research could pave the way for tailored interventions that could have the potential to substantially alleviate the cardiovascular burden associated with T2DM.

Aims and objectives: To assess the cardiovascular risk and its determinants among T2DM patients.

Methods: A cross sectional study was conducted among known diabetes patients accessing urban outreach clinic serving approximately 20,000 population across 18 urban slums in central Karnataka from September 2022 to June 2023. A pre-tested semi-structured questionnaire was used to collect information on socio-demographic details and CVD risk was assessed using QRISK3 score. Data were entered in Excel 2019 (Microsoft, Redmond, WA, USA) and analysed using SPSS version 25.0 (IBM Corp., Armonk, NY, USA) and are presented in the tables and figures.

Results: A total of 483 adults above 30 years participated in the study. Among them, the majority were men (67.9%). Cardiovascular risk factors were found more among males and the 10-year cardiovascular risk assessment prediction through QRISK3 score was higher among males compared to females and it was found to be statistically significant (13.5±8.6% vs. 19.5±10.1%, p<0.001).

Conclusion: According to a comparison of cardiovascular risk variables by gender among diabetic patients using the QRISK3's 10-year risk assessment, males, smokers/tobacco users, obese, and known hypertensives had significantly greater risk.

## Introduction

Cardiovascular disease (CVD) causes an estimated 17.7 million deaths every year. These deaths consist primarily of ischemic heart disease and stroke. India accounts for over one-fifth of premature cardiovascular deaths worldwide [1]. The burden of type 2 diabetes mellitus (T2DM) and CVD is staggering and ever-increasing. According to the International Diabetes Federation (IDF), in 2021, approximately 537 million adults were living with diabetes, and this number is projected to rise to 642 million by 2040 [2]. Concurrently, cardiovascular diseases, including coronary artery disease, stroke, and heart failure, remain the leading causes of morbidity and mortality worldwide [1].

The Global Burden of Disease Study estimated that in 2019, cardiovascular diseases were responsible for 17.9 million deaths, accounting for nearly a third of all global deaths [3]. T2DM and CVD represent two intertwined and escalating global health challenges [4]. Over the past few decades, both conditions have witnessed an alarming surge in prevalence, with significant implications for individual health, healthcare systems, and economies worldwide [5,6].

In recent years, there has been a growing body of research shedding light on the intricate mechanisms underlying the link between T2DM and CVD. These studies have revealed not only the pathophysiological connections but also underscored the urgency of targeted intervention strategies for individuals with diagnosed T2DM [7-9]. The focus on diagnosed patients is crucial, as this early phase represents a window of opportunity for implementing preventive measures that could significantly mitigate the cardiovascular burden associated with T2DM.

Epidemiological evidence has consistently demonstrated that individuals with T2DM face a significantly elevated risk of developing CVD [10]. The risk is further exacerbated by a multitude of factors, including obesity, dyslipidemia, hypertension, and insulin resistance, all of which commonly coexist with T2DM [4].

The Framingham Heart Study and other landmark investigations have highlighted the strong association between T2DM and adverse cardiovascular outcomes, making it clear that diabetes represents a major independent risk factor for CVD development [11,12].

With this background, the current study on assessment of cardiovascular risk factors among people living with T2DM was conducted.

## Materials and methods

A comparative analytical study was conducted among detected people with T2DM attending an urban outreach clinic in the months of September 2022 to June 2023. The outreach clinic of the tertiary care teaching hospital caters to approximately 20,000 urban slum population. The Institutional Ethics Committee of Jagadguru Jayadeva Murugarajendra Medical College, Davangere, issued approval JJMMC/IEC/29/2022. Diagnosed T2DM patients aged above 30 years residing in the urban catchment area attending the outreach clinic were eligible to participate in the study. Data collection was conducted for seven months from September 2022 to March 2023. Participants with pre-existing cardiovascular diseases like heart attack, angina, stroke, or transient ischemic attack were excluded from the study. For the study, universal sampling was carried out, all the eligible participants attending the outreach clinic were offered to be part of the study, and were included after informed written consent.

All the participants underwent detailed clinical assessments, including anthropometric measurements (height, weight, waist circumference, and blood pressure), random glucose levels, and lipid profile.

A pre-tested semi-structured questionnaire was used to collect information on basic socio-demographic profiles and cardiovascular risk factors such as smoking history, alcohol consumption, physical activity levels, dietary habits, family history of cardiovascular diseases, and medication use, also on their medical history, duration of diabetes, socio-demographic characteristics, and access to healthcare services of the participants.

Body mass index (BMI) was calculated using Quetelet’s formula [13] and WHO BMI classification for the Asian adult population was used to categorize the study participants [14].

Cardiovascular risk assessment was computed using a prediction algorithm for cardiovascular disease, the QRISK3 chart since, in the opinion of the Lipid Association of India (LAI), it is the most appropriate for Indians. The QRISK3 algorithm determines a person's 10-year risk of having a heart attack or stroke. The higher the score, the greater the risk. The algorithms in QRISK3 quantify the absolute risks of cardiovascular disease in people aged 25-84 years, which include established risk factors and new risk factors including chronic kidney disease, migraine, corticosteroid use, systemic lupus erythematosus (SLE), atypical antipsychotic use, severe mental illness, erectile dysfunction, and a measure of blood pressure variability. The advantage of QRISK3 over other prediction models is that it considers diverse ethnicities, including Indians, making it possible to assess the overall cardiovascular risk of the Indian population [15].

Age ≥45 years in men and ≥55 years in women, hypertension, smoking or tobacco use, and low high-density lipoprotein cholesterol (HDL-C) (40 mg/dL in men and 50 mg/dL in women) are the major atherosclerotic cardiovascular disease (ASCVD) risk variables as defined by LAI considered in the current analysis. Study participants with diabetes and two or more of the above-mentioned major risk factors and evidence of target organ damage were grouped as very high risk as per LAI guidelines [16].

Socio-demographic and clinical data were analysed by calculating the mean (±SD) in case of continuous variables and the absolute (n) and relative (%) frequency in case of categorical variables. For categorical variables, differences across groups were evaluated using Chi-square tests. A p-value of <0.05 was considered significant. Analyses were performed using SPSS version 25.0 for Windows (IBM Corp., Armonk, NY, USA); for calculating QRISK-score, the QRISK3 package was used.

## Results

A total of 483 adults were part of the study. Among them, the majority were males (68%) and belonged to the age group 30-44 years with an average age of 40.4 ± 9.2 years. The majority belonged to class III of the modified BG Prasad socioeconomic status (SES) classification updated for November 2022. The mean duration of diabetes among the participants was 6.3 ± 2.4 years. Males were taller (156.8 ± 9.80 cm) and heavier (72.3 ± 7.14 kg) compared to females (height: 146.5 ± 11.23 cm, weight: 59.9 ± 9.21 kg) and females had higher BMI (26.3 ± 3.18 kg/m2) as compared to males (21.1 ± 3.61 kg/m2). Mean values for central adiposity (waist circumference (WC) and waist-hip ratio) were also higher among females than males, while the cardiometabolic risk factors, systolic blood pressure (SBP) (144.7 ± 12.80 mmHg), and random glucose (262.3 ± 32.70 mg/dL), were higher among males compared to females (118.3 ± 20.90 and 212.8 ± 24.48 respectively) (Table 1).

**Table 1:** Gender-wise clinical profile of the study participants SD: standard deviation

Variables	Total (N = 483) Mean ± SD	Male (N = 328) Mean ± SD	Female (N = 155) Mean ± SD	t value	p value
Age (years)	40.4 ± 9.2	42.3 ± 8.1	38.6 ± 9.9	4.355	<0.0001
Duration of diabetes mellitus (DM: years)	6.3 ± 2.4	5.9 ± 4.3	6.1 ± 3.8	80.909	<0.0001
Height (cm)	152.9 ± 8.6	156.8 ± 9.80	146.5 ± 11.23	10.280	<0.0001
Weight (Kg)	69.6 ± 5.3	72.3 ± 7.14	59.9 ± 9.21	16.181	<0.0001
Body mass index (BMI: Kg/m2)	24.8 ± 3.4	21.1 ± 3.61	26.3 ± 3.18	15.339	<0.0001
Waist circumference (cm)	86.9 ± 9.3	85.8 ± 9.1	89.7 ± 8.3	4.520	<0.0001
Waist hip ratio (WHR)	0.96 ± 0.06	0.95 ± 0.05	0.98 ± 0.08	0.041	0.9673
Systolic blood pressure (SBP: mmHg)	129.8 ± 14.70	144.7 ± 12.80	118.3 ± 20.90	17.088	<0.0001
Diastolic blood pressure (DBP: mmHg)	85.30 ± 14.85	89.30 ± 11.45	79.70 ± 16.85	7.341	<0.0001
Random glucose level (RBS: mg/dl)	240.50 ± 27.63	262.3 ± 32.70	212.8 ± 24.48	16.754	<0.0001

Males had more ASCVD risk factors compared to females. Men also had a higher risk than females because they were more likely to smoke or use tobacco (62.6% vs. 19.1%), have hypertension (63.8% vs. 33.7%), and be older (age 45 is considered a major risk factor for males, whereas this is from age 55 for females) (Table 2).

**Table 2:** Atherosclerotic cardiovascular (ASCVD) risk factors of the study participants

Variables	Total N = 483 (%)	Male N = 328 (%)	Female N = 155 (%)	p value
Age (years) as risk factor				0.03
Male <45, female <55	246 (69.6)	139 (42.5)	107 (69.2)	
Male ≥45, female ≥55	147 (30.4)	99 (57.5)	48 (30.8)	
High-density lipoprotein (HDL: mg/dl)				<0.0001
Normal	152 (31.5)	71 (52.4)	81 (21.6)	
Low (males <40, females <50)	331 (68.5)	257 (47.6)	74 (78.4)	
Smoking/Tobacco use				<0.00018
No	247 (51.2)	122 (37.4)	125 (80.9)	
Yes	236 (48.8)	206 (62.6)	30 (19.1)	
Hypertension				<0.0001
No	222 (46.0)	119 (36.2)	103 (66.3)	
Yes	261 (54.0)	209 (63.8)	52 (33.7)	
Cholesterol-lowering treatment				0.09
No	429 (88.8)	286 (87.3)	143 (92.7)	
Yes	54 (11.2)	42 (12.7)	12 (7.3)	
Anti-hypertension treatment				0.0001
No	309 (64.0)	191 (58.2)	118 (76.1)	
Yes	174 (36.0)	137 (41.8)	37 (23.9)	

Based on the QRISK3 risk chart the diagnosed T2DM patients had an average QRISK3-score of 17.1 ± 9.9% with females having a lower risk than males (13.5 ± 8.6% vs. 19.5 ± 10.1%, p < 0.0001). Also, smokers had the highest CV risk, followed by obesity, hypertension, and male gender (Table 3).

**Table 3:** QRISK3 score (mean with SD) of the study participants *Hypertension based on anti-hypertension treatment SD: standard deviation

Females (N = 155) Mean ± SD	Males (N = 328) Mean ± SD	p value
13.5 ± 8.6	19.5 ± 10.1	<0.0001
Non-smokers/tobacco users (N = 247) Mean ± SD	Smoker/Tobacco user (N = 236) Mean ± SD	
12.8 ± 9.3	23.3 ± 11.9	<0.0001
Normotension (N = 309) Mean ± SD	Hypertension* (N = 174) Mean ± SD	
13.7 ± 11.9	21.6 ± 18.2	<0.0001
Normal (N = 292) Mean ± SD	Obese (N = 191) Mean ± SD	
13.1 ± 10.6	21.8 ± 18.7	<0.0001

## Discussion

The present study has investigated the 10-year estimated cardiovascular risk among people living with diabetes mellitus and their probable determinants using QRISK3 score. According to this score, smokers/tobacco users 10% more CV risk compared to non-smokers/tobacco users, obese had almost 9% higher risk than participants with normal BMI, hypertensives had 8% higher risk compared to normotensives and males had 6% higher risk than females.

A similar multi-centric study [17] conducted among newly detected type 2 diabetics in 121 cities throughout 27 Indian states found 7% higher CV risk among smokers followed by 5% higher risk among hypertensives on QRISK3 scores. Another study [18] conducted among a large Mediterranean sample of type 2 diabetes patients using European High-Risk Chart (ESC) found majority of the males with T2DM had very high risk for CVD events compared to females (53.4% vs 50.7%).

High risk of CVD events was also found among males with T2DM in the present study which may be because of higher ASCVD risk among Indians compared to the Caucasian population, also possibly because of the stricter classification of the Lipid Association of India when compared to the ESC classification. Indian studies [17,19] also show being an Indian male and older than 45 is already a big risk factor according to LAI, however according to ESC standards this is a decade later. Indians get coronary artery disease one or two decades earlier than their Western counterparts [19]. Additionally, the QRISK3 score assigns the Indian population a greater risk rating than both whites (3% higher) and black Africans (7% higher) [15].

Another large sample study [20] looking at five-year CVD risk among adults residing in urban slums in Karnataka using National Health and Nutrition Examination Survey CVD risk assessment charts found that participants who were currently unmarried, illiterate, and unemployed were significantly associated with higher CVD risk. In the present study also CVD risk was found higher among minority populace, with fewer years of education, involved in unskilled occupations, and belonging to joint family, but a significant association was found only for smokers, hypertensives, obese, and males.

In the current study, more than half of our study participants (54%, n = 483) were known to be hypertensive; this is on par with some of the studies that reported 60% prevalence of hypertension among diabetics. Another multi-centre study [17] conducted among known diabetics found a 42% prevalence of hypertension. The current study participants were relatively young (40.4 ± 9.2 years) and the majority were on treatment (66.6%). During the blood pressure measurement conducted during participant’s visit to the urban outreach clinic, mean SBP was 129.8±14.70 mmHg. Studies [21-22] have shown type 2 diabetes patients often have elevated night-time blood pressure, and health education on identification and treatment seeking is needed in this population.

According to the LAI guidelines, obesity is not a major risk factor but still needs lifestyle alteration. According to the QRSIK3 score assessment, among the study participants, obese patients had an 8.7% higher risk compared with those with normal BMI. However, studies [23,24] have shown BMI also has some limitations as a risk predictor thus it may be questioned if it is appropriate to determine CVD risk based on BMI values for the current study population. Indians have a body composition that differs from other ethnicities so that BMI may not be the best method to estimate CVD risk in Indians. Using body fat measurement indices like waist circumference, waist-to-hip ratio and waist-to-height ratio should be preferred to estimate the CVD risk [25].

Of all participants, 48.8% were smokers/tobacco users and it was higher among the male population. Tobacco use is one of the major risk factors for ASCVD and for onset of DM in this population. The QRISK3 score assessment considers smoking use as an important factor, and LAI considers tobacco use also during risk assessment. Compared to developed countries, use of chewing/smokeless tobacco is higher among the Indian population [25]. Studies [26,27] have shown smokeless tobacco contains a higher amount of nicotine compared to cigarettes and is one of the contributors to insulin resistance [28,29]. There is a need to educate the community on the harmful effects of using smokeless tobacco.

## Conclusions

The present study conducted among an urban slum population found that the majority of the people living with T2DM had higher cardiovascular risk factors and it was more among males compared to females. Risk was also found to be high among smokers, obese and hypertensive. Based on the findings of the Cardiovascular Risk Assessment among diagnosed type 2 diabetics in urban slums of Karnataka, India, there is a need to address the identified cardiovascular risk factors and improve the overall cardiovascular health of this population. Early diabetes detection and management, access to health education and awareness on lifestyle modifications, appropriate pharmacological interventions, multi-disciplinary approach that addresses both diabetes management and cardiovascular risk identification and reduction are the need of the hour. Healthcare providers, policymakers and community stakeholders need to work together to reduce cardiovascular risk among diabetes patients and thus improve cardiovascular outcomes and the overall well-being of the population. 

This research will provide valuable insights into the cardiovascular risk profile of diagnosed type 2 diabetes patients in urban slums of Karnataka. The findings will contribute to the understanding of the early cardiovascular risk factors in this vulnerable population and may inform targeted interventions and policies to reduce the burden of cardiovascular diseases in urban slum settings. This could also act as a pilot study for larger community-based studies.
